# Genito-pelvic pain from a couples’ perspective

**DOI:** 10.1080/24740527.2018.1452499

**Published:** 2018-05-21

**Authors:** Natalie O. Rosen

**Affiliations:** Dalhousie University

Vulvodynia is a genito-pelvic pain condition that is characterized by recurrent vulvo-vaginal pain that does not have an identifiable cause. With a prevalence of 8% to 12% in the general population, this pain adversely affects women’s and their partners’ psychological, relationship, and sexual well-being. The pain interferes with both sexuality and one’s romantic relationship, making interpersonal variables especially relevant to this condition. In this presentation, Dr. Rosen will review her CIHR-funded research focusing on novel interpersonal predictors of adjustment to vulvodynia. Using daily diaries and observational study designs, she will first present her findings on the role of partner responses to the pain, intimacy, and sexual goals (i.e., the reasons for having sex) in the pain and psychosexual impairments of vulvodynia. Results from these studies were used to co-develop a novel, cognitive-behavioural couple therapy (CBCT) for women with vulvodynia and their partners. Dr. Rosen will next provide an overview of this intervention and the results of a pilot study, which found significant pre- to post-treatment improvements in the primary outcome (pain during intercourse), as well as several psychosexual outcomes for both women and partners, including sexual function, sexual satisfaction, and pain catastrophizing. She is currently conducting a two-centre randomized clinical trial to evaluate the efficacy of CBCT by testing it against a standard medical treatment – lidocaine. Overall, this program of research contributes to providing much needed empirically validated psychological interventions for genito-pelvic pain from a couples’ perspective

